# Red blood cell distribution width-to-albumin ratio as a novel predictor for mortality in breast cancer patients admitted to ICU: a retrospective analysis using MIMIC-IV 3.1

**DOI:** 10.1186/s12911-026-03711-x

**Published:** 2026-07-13

**Authors:** Chenyan Hong, Ke Yin, Shenchao Guo, Jin Luo

**Affiliations:** Department of Thyroid and Breast Surgery, The First Affiliated Hospital of Ningbo University, Ningbo, China

**Keywords:** Red blood cell distribution width, Albumin, Prognostic, Breast cancer, 1-Year mortality

## Abstract

**Background:**

The red blood cell distribution width-to-albumin ratio (RAR), an emerging biomarker integrating inflammation and nutritional status, has not been systematically evaluated for its prognostic value in breast cancer patients admitted to intensive care units (ICU).

**Methods:**

We conducted a retrospective cohort study using data from the MIMIC-IV 3.1 database, including 881 adult breast cancer patients admitted to the ICU. Patients were stratified into high- and low-RAR groups based on maximally selected rank statistics. Kaplan–Meier analysis, multivariable Cox proportional hazards models, restricted cubic splines, subgroup analysis, time-dependent concordance index (C-index) curves and Boruta feature selection (iterative random forest with shadow feature comparison) were applied to assess the association between RAR and 1-year all-cause mortality. Robustness was examined using E-value and propensity score weighting methods.

**Results:**

Patients with high RAR (> 4.96) had significantly higher 1-year mortality compared to those with low RAR (log-rank *P* < 0.001). In adjusted models, high RAR independently predicted mortality (HR = 1.65, 95% CI: 1.33–2.06). Across the four models, E-values for the point estimates ranged from 2.18 (fully adjusted model, HR 1.65) to 2.82 (unadjusted model, HR 2.19); the E-value for the lower confidence limit of the primary model was 1.73. Each standard deviation increase in RAR was associated with a 17% higher mortality risk (HR = 1.17, 95% CI: 1.09–1.25). Restricted cubic spline analysis demonstrated a linear dose–response relationship (P for nonlinearity > 0.05). Incorporating RAR into SOFA and APS III scores improved prognostic performance (*P* < 0.001). Feature importance ranking further highlighted RAR as a major predictor. Sensitivity analyses using overlap-weighting (HR = 1.56, 95% CI: 1.25–1.96) and matching-weighting (HR = 1.53, 95% CI: 1.22–1.93) confirmed robustness.

**Conclusion:**

RAR is an easily obtainable biomarker derived from routine laboratory testing that may enhance risk stratification for ICU-admitted breast cancer patients. Its integration into prognostic models may facilitate early decision support.

**Supplementary Information:**

The online version contains supplementary material available at https://doi.org/10.1186/s12911-026-03711-x.

## Introduction

Breast cancer remains one of the most prevalent malignancies among women worldwide, representing a significant global health concern. According to GLOBOCAN 2020, breast cancer accounted for approximately 2.3 million new cases and 685,000 deaths globally, making it the most frequently diagnosed cancer and a leading cause of cancer-related mortality in women [1]. The increasing incidence and high treatment costs have placed a substantial economic burden on healthcare systems, particularly in low- and middle-income countries where access to timely diagnosis and treatment is limited [2]. Despite advances in therapeutic strategies, predicting prognosis in breast cancer patients remains challenging. Traditional prognostic tools often rely on histopathological features and advanced molecular profiling, which may not be readily available or cost-effective in routine clinical practice. Therefore, there is a growing need for simple, accessible, and inexpensive biomarkers to aid in prognostication and guide personalized treatment.

The red cell distribution width-to-albumin ratio (RAR) has garnered growing attention as a prognostic biomarker reflecting the interplay between inflammation and nutritional status. While red cell distribution width (RDW) [3–5] and serum albumin [6–8] have been individually associated with survival outcomes, their combined ratio may offer improved prognostic utility due to its integration of two distinct but complementary physiological domains. In population-based studies, elevated RAR has been linked to increased risks of mortality and morbidity, particularly in older adults and patients with chronic conditions, where systemic inflammation and hypoalbuminemia frequently coexist [9]. Lu et al., utilizing data from the MIMIC-III database, demonstrated that an elevated RAR is independently associated with increased all-cause mortality among cancer patients [10]. RAR captures subtle shifts in erythropoiesis and protein metabolism that may not be apparent when RDW or albumin are assessed alone. In the context of oncology, and breast cancer specifically, this ratio may serve as a surrogate for tumor-induced systemic changes. Breast cancer progression is known to be influenced by a pro-inflammatory tumor microenvironment and host nutritional status, both of which are reflected in this composite index. Unlike molecular techniques or pathological features, RAR is inexpensive, routinely available, and does not require specialized equipment, making it particularly valuable in resource-limited settings.

Machine learning (ML), as a major field within artificial intelligence, demonstrates the capacity to extract key features from complex, multidimensional datasets. Consequently, it has found widespread application in creating diagnostic and predictive models for the accurate identification of numerous diseases [11]. The RandomForest algorithm is an ensemble learning technique that improves predictive accuracy and robustness through the aggregation of outputs from multiple decision trees, each trained on a distinct subset of samples. This decision tree-based method enabled the identification of the most influential variables [12]. Compared to conventional single-model approaches, Random Forest can accommodate high-dimensional covariate spaces, capture non-linear relationships and complex variable interactions without prior specification, and provide data-driven rankings of predictor importance, thereby facilitating the identification of clinically informative biomarkers [13].

To date, no studies have investigated the prognostic significance of RAR in breast cancer. Given that both elevated RDW and hypoalbuminemia have been independently associated with systemic inflammation and poor outcomes in various malignancies, exploring the potential correlation between RAR and breast cancer prognosis is warranted. Establishing such a relationship could offer a novel, cost-effective biomarker for risk stratification and treatment decision-making in clinical practice.

## Materials and methods

### Study population

This retrospective analysis was conducted using health-related data extracted from version 3.1 of the MIMIC-IV database from 2008 to 2022. The MIMIC- IV database is a large, publicly available critical care database that contains health data from intensive care unit (ICU) admissions, including patient demographics, vital signs, laboratory results, and clinical outcomes. The data is fully de-identified and its use does not impact the clinical treatment of the patients. Accordingly, this study complied with the ethical principles of the Declaration of Helsinki and was granted a waiver of institutional ethical approval and informed consent.

**Fig. 1:**
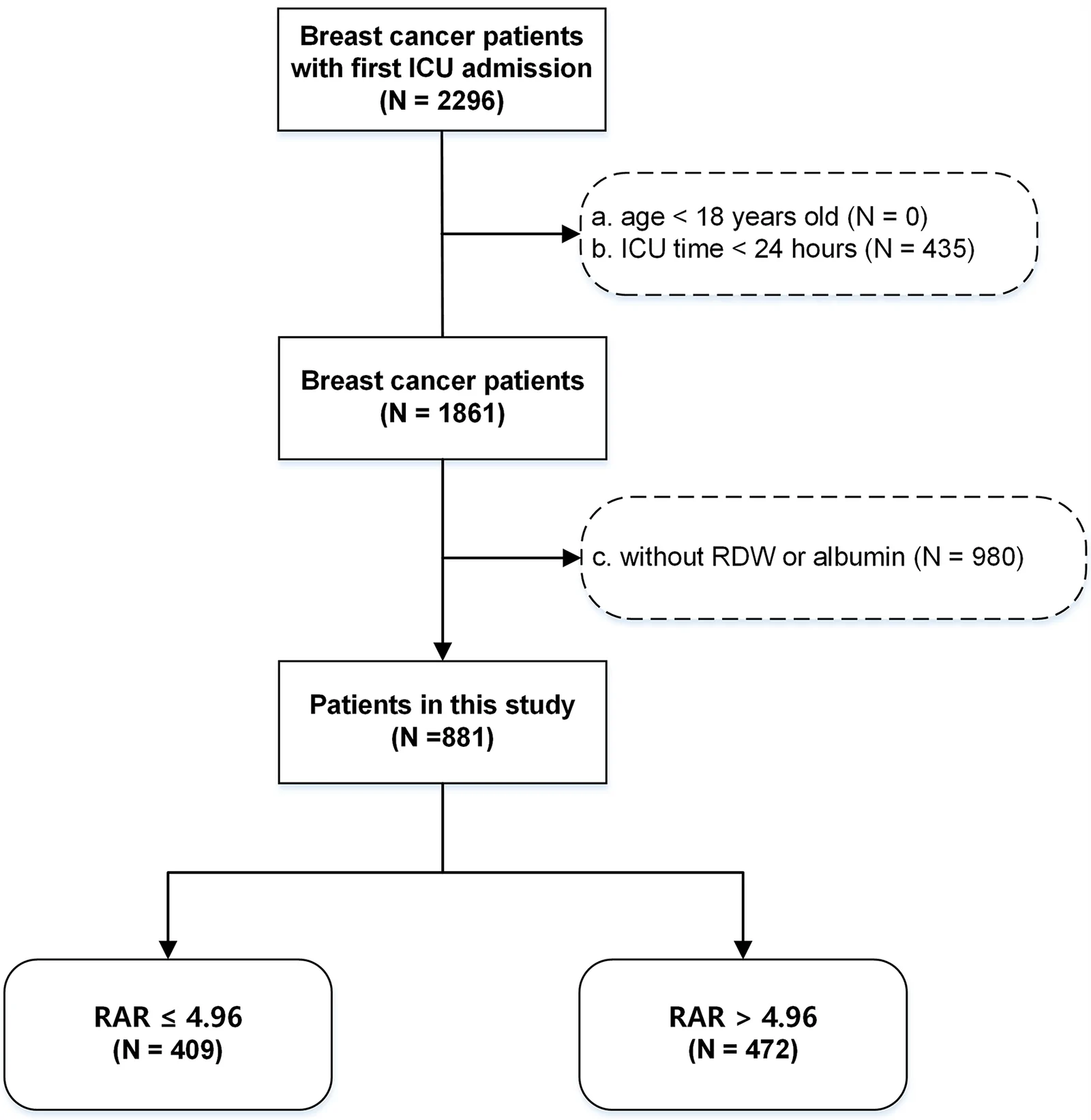
Flowchart of patient eligibility, inclusion, and exclusion criteria. Abbreviations: ICU: Intensive Care Unit; RDW: red cell distribution width

Breast cancer patients were identified from the MIMIC-IV database according to diagnostic codes based on the 9th and 10th revisions of the International Classification of Diseases (ICD-9 and ICD-10). Only a minimum ICU stay threshold of ≥ 24 h was applied; no upper limit on length of stay was imposed, ensuring that patients with prolonged ICU courses were not excluded. For individuals with multiple ICU admissions, only data from the first admission were included in the analysis. The exclusion criteria were as follows: (a) age under 18 years at the time of initial ICU admission; (b) missing RDW or albumin values on the first day of admission; and (c) ICU stay of less than 24 h. A total of 881 eligible patients were ultimately included and stratified into two groups according to RAR (Fig. 1).

### Data collection

The following variables were primarily included in this study: demographic characteristics (age, gender), vital signs [respiratory rate, heart rate, systolic blood pressure, diastolic blood pressure, saturation of peripheral oxygen (SpO2)], comorbidities [hypertension, diabetes, chronic kidney disease, acute kidney injury (AKI) stage, heart failure, cirrhosis, sepsis, COVID-19], laboratory tests [white blood cell (WBC), hemoglobin, red cell distribution width (RDW), platelets, alanine aminotransferase (ALT), aspartate aminotransferase (AST), total bilirubin, albumin, creatinine, blood urea nitrogen (BUN), glucose, anion gap, lactate dehydrogenase], disease severity scores [Sequential Organ Failure Assessment (SOFA) score, Acute Physiology Score III (APS III), Oxford Acute Severity of Illness Score (OASIS), Charlson Comorbidity Index (CCI)], continuous renal replacement therapy (CRRT), invasive ventilation, one-year mortality. The above variables obtained during the first 24 h following ICU admission were extracted for analysis. RAR was calculated using the following formula: RAR = red cell distribution width (RDW, %) divided by serum albumin (g/dL). The primary endpoint of the study was all-cause mortality within one year following hospital discharge.

### Statistical analysis

All statistical analyses were performed using R software (version 4.3.1), with a significance level of *p* < 0.05. Continuous variables are expressed as mean±standard deviation (SD) or median with interquartile range (IQR), while categorical variables are presented as frequency and percentage (%).

The optimal cut-off value of RAR was obtained for early warning of mortality risk, under the maximally selected rank statistics using the survminer package. To assess potential optimism arising from the data-derived threshold, the cut-off selection via maximally selected rank statistics and the subsequent Cox model were subjected to 1000 bootstrap resamples. We report the range (or interquartile range) of selected cut-off values and the optimism-corrected hazard ratio with 95% confidence interval, following similar bootstrap-validation approaches in ratio-based biomarker studies [14, 15]. Based on this cutoff, patients were categorized into high- and low-RAR groups. Kaplan–Meier survival curves and Log rank test were subsequently constructed for each group. Baseline characteristics between groups were compared using the Mann–Whitney U test for non-normally distributed continuous variables, the independent t-test for normally distributed continuous variables, and the chi-square test for categorical variables.

Regarding RAR as a continuous (increase per unit) or categorical variable, cox regression models with varying degrees of adjustment for potential confounders were employed to estimate hazard ratios (HRs) and 95% confidence intervals (CIs) for all-cause mortality. The selection of covariates was based on prior literature and clinical expertise, rather than data-driven approaches [16]. In Model 0, only RAR was included without any adjustment for covariates. Model 1 adjusted for age, gender, diabetes, hypertension, AKI stage, CKD, heart failure, sepsis3, cirrhosis and COVID-19 in patients. Model 2 adjusted for the covariates in Model 1 and WBC, hemoglobin, AST, total bilirubin, creatinine, BUN, anion gap, LDH, CRRT and ventilation. Model 3 adjusted for the covariates in Model 2, as well as SOFA, APS Ⅲ, OASIS and CHARLSON. Considering the MIMIC database lacks certain prognostic factors (e.g. tumor pathological staging), E-Value was calculated using the EValue package to assess potential unmeasured confounding [14]. The E-value for a given risk or rate ratio (RR) was calculated as: $\:E\:value=RR+\sqrt{RR\times\:\left(RR-1\right)}\:\:$). For the confidence interval, the same formula was applied to the limit closest to the null (set to 1 if the interval crossed it). Because 1-year mortality is a common outcome, hazard ratios were first converted to approximate risk ratios before computing the E-value, following the approach implemented in the E-value R package. To avoid model overfitting, the variance inflation factor (VIF) was used to assess multicollinearity among all included covariates (Supplementary Fig. 1). Based on Model 3, restricted cubic spline analysis was employed to characterize the dose–response relationship between RAR and all-cause mortality, with threshold effect analysis conducted when appropriate [15]. Moreover, we conducted additional subgroup analyses and mediation analysis by adjusted Cox models.

Furthermore, a time-concordance index curve was generated to assess the incremental prognostic value of RAR for mortality. The Boruta feature selection algorithm (a wrapper around the random-forest classifier) was applied to assess the relative importance of RAR and other baseline variables in predicting 1-year all-cause mortality. At each iteration, it creates ‘shadow’ features by randomly permuting copies of all real predictors, fits a random forest on the combined set, and retains only those real predictors whose importance significantly exceeds the maximum importance among the shadow features across all iterations. Because random forests can distribute importance among strongly correlated predictors, the resulting ranking should be interpreted qualitatively rather than as a strict unique ordering. Finally, to enhance the robustness of the analysis results, sensitivity analyses were conducted using two alternative propensity score (PS) weighting approaches: overlap-weighting and matching-weighting.

## Results

### Patient characteristics

**Fig. 2:**
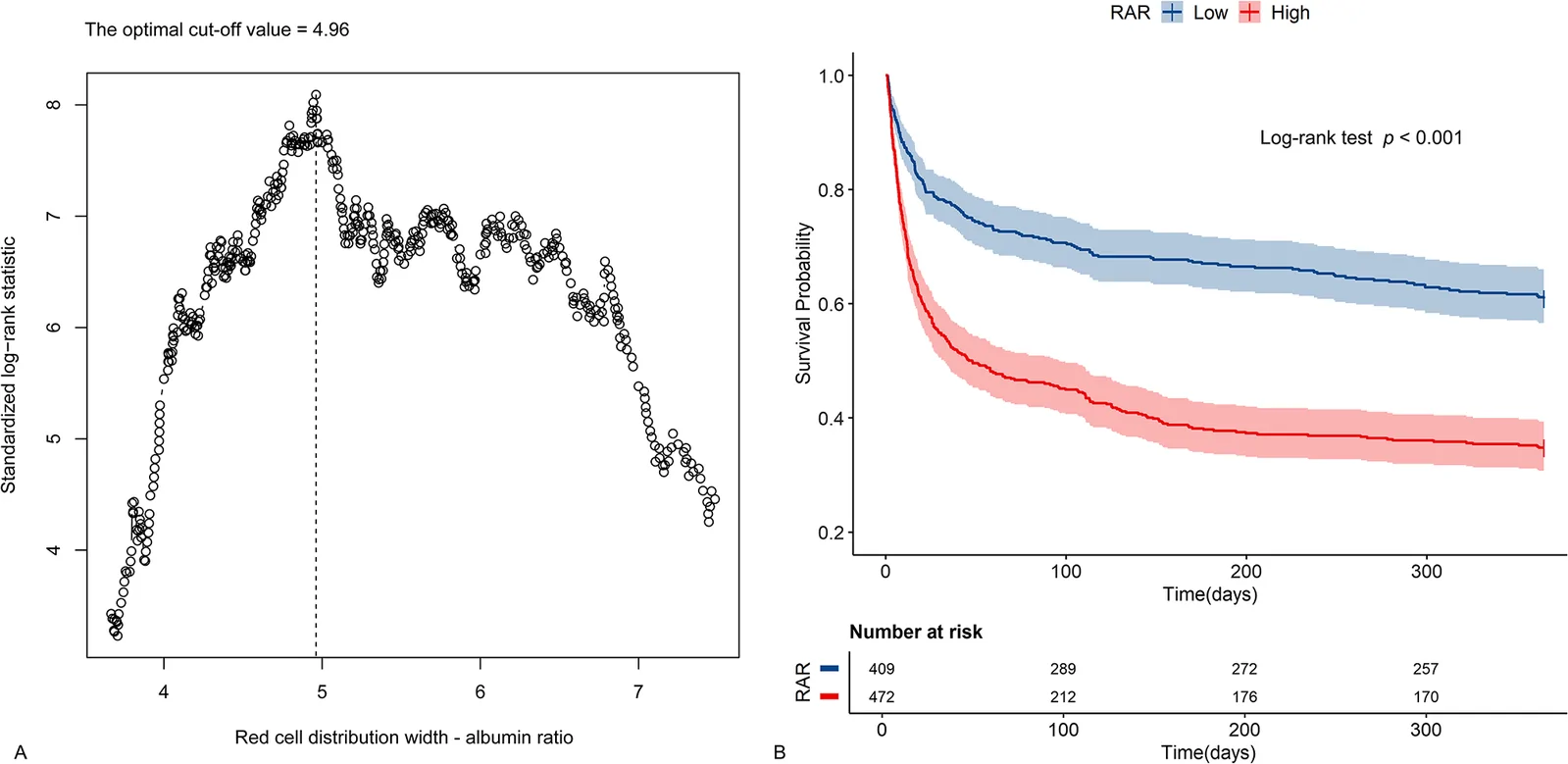
(**A) **Identification of the optimal RAR cutoff value using the standardized log-rank statistic. (**B**)Comparison of the cumulative survival probability between the high and low RAR groups

A total of 881 patients with breast cancer were ultimately enrolled in this study. Based on an optimal cutoff value of 4.96 for the RAR (Fig. 2A), patients were stratified into the high RAR group (*n* = 472) and the low RAR group (*n* = 409) (Fig. 1). The clinical characteristics differed partially between the two groups. Notably, although patients in the high RAR group were younger than those in the low RAR group, they exhibited a higher 1-year all-cause mortality rate (Table 1).

**Table 1:** Baseline information according to the levels of red cell distribution width - albumin ratio

Characteristics	Overall *n* = 881	Low (≤ 4.96) *n* = 409	High (> 4.96) *n* = 472	*p* value
age (years)	74.04 ± 12.40	75.35 ± 12.40	72.90 ± 12.30	0.003
male	13 (1.5%)	11 (2.7%)	2 (0.4%)	0.005
respiratory rate (/min)	20.23 ± 6.26	19.57 ± 6.22	20.81 ± 6.25	0.003
heart rate (/min)	90.73 ± 20.56	86.20 ± 17.99	94.66 ± 21.81	< 0.001
systolic blood pressure (mmHg)	123.23 ± 25.47	127.43 ± 25.44	119.60 ± 24.96	< 0.001
diastolic blood pressure (mmHg)	68.37 ± 19.22	70.10 ± 19.64	66.88 ± 18.74	0.013
saturation of peripheral oxygen (%)	98.00 (95.00 100.00)	98.00 (95.00 100.00)	97.00 (94.00 100.00)	0.069
hypertension	385 (43.7%)	198 (48.4%)	187 (39.6%)	0.009
diabetes	263 (29.9%)	122 (29.8%)	141 (29.9%)	0.989
chronic kidney disease	162 (18.4%)	70 (17.1%)	92 (19.5%)	0.364
AKI stage				< 0.001
0	285 (32.3%)	169 (41.3%)	116 (24.6%)	
1	108 (12.3%)	64 (15.6%)	44 (9.3%)	
2	257 (29.2%)	114 (27.9%)	143 (30.3%)	
3	231 (26.2%)	62 (15.2%)	169 (35.8%)	
heart failure	298 (33.8%)	126 (30.8%)	172 (36.4%)	0.078
cirrhosis	52 (5.9%)	11 (2.7%)	41 (8.7%)	< 0.001
sepsis	490 (55.6%)	193 (47.2%)	297 (62.9%)	< 0.001
COVID-19	13 (1.5%)	8 (2.0%)	5 (1.1%)	0.271
white blood cell (10^9/L)	10.60 (7.40 15.20)	10.40 (7.50 14.30)	11.05 (7.38 16.30)	0.115
hemoglobin (g/dL)	10.24 ± 2.01	11.03 ± 1.89	9.56 ± 1.87	< 0.001
red cell distribution width (%)	15.68 ± 2.57	14.15 ± 1.30	17.00 ± 2.67	< 0.001
platelets (10^9/L)	201.00 (139.00 276.00)	212.00 (155.00 272.00)	193.50 (119.50 281.25)	0.015
alanine aminotransferase (U/L)	23.00 (14.00 44.00)	23.00 (14.00 41.00)	23.00 (13.00 45.25)	0.964
aspartate aminotransferase (U/L)	34.00 (22.00 66.00)	31.00 (21.00 57.00)	36.00 (23.00 82.00)	0.010
total bilirubin (mg/dl)	0.60 (0.40 1.00)	0.50 (0.30 0.80)	0.70 (0.40 1.30)	< 0.001
albumin (g/dl)	3.05 ± 0.58	3.48 ± 0.43	2.68 ± 0.44	< 0.001
creatinine (mg/dl)	0.90 (0.70 1.50)	0.90 (0.60 1.20)	1.00 (0.70 1.80)	< 0.001
blood urea nitrogen (mg/dl)	21.00 (14.00 35.00)	18.00 (12.00 28.00)	24.00 (16.00 40.00)	< 0.001
glucose (mg/dL)	128.00 (102.00 167.00)	130.00 (106.00 172.00)	127.00 (100.00 163.00)	0.031
anion gap (mmol/L)	14.83 ± 4.42	14.75 ± 3.91	14.91 ± 4.83	0.596
lactate dehydrogenase (U/L)	270.00 (205.00 395.00)	261.00 (203.00 365.00)	284.00 (206.50 429.00)	0.009
SOFA	4.95 ± 3.33	4.03 ± 3.03	5.75 ± 3.38	< 0.001
APS Ⅲ	48.49 ± 20.11	42.75 ± 18.84	53.46 ± 19.87	< 0.001
OASIS	33.33 ± 8.59	31.96 ± 8.69	34.51 ± 8.32	< 0.001
CHARLSON	7.03 ± 2.77	6.59 ± 2.71	7.40 ± 2.77	< 0.001
continuous renal replacement therapy	43 (4.9%)	9 (2.2%)	34 (7.2%)	< 0.001
invasive ventilation	282 (32.0%)	111 (27.1%)	171 (36.2%)	0.004
one-year mortality	468 (53.1%)	160 (39.1%)	308 (65.3%)	< 0.001

### Survival analysis

According to the Kaplan–Meier curves, patients with a high RAR demonstrated a significantly higher all-cause mortality rate (log-rank *P* < 0.001, Fig. 2B).

Four Cox regression models were constructed to evaluate the prognostic impact of RAR in this population (Table 2). The variance inflation factors (VIFs) for all variables included in the models were less than 5 (Supplementary Figure. 1), indicating no significant multicollinearity. In Model 3, after full adjustment for covariates, the high RAR group (compared with the low RAR group) had a hazard ratio of 1.65 (95% CI: 1.33–2.06) for 1-year all-cause mortality. Similar results were observed in Model 0 (HR:2.19, 95% CI: 1.81–2.65), Model 1 (HR:1.95, 95% CI: 1.59–2.38), and Model 2 (HR:1.83, 95% CI: 1.47–2.27). When RAR was analyzed as a continuous variable, each standard deviation increase in RAR was associated with a 17% higher risk of all-cause mortality after multivariable adjustment (HR:1.17, 95% CI: 1.09–1.25) (Table 2). Across the four models, all E-values were greater than 2, indicating that only unmeasured confounders with risk ratios of at least the corresponding E-values could fully account for the observed associations, thereby reinforcing the robustness of our results. Meanwhile, Bootstrap validation (1000 resamples) yielded a median cut-off of 4.96 (IQR: 4.79 to 5.02), with an optimism-corrected HR of 1.52 (95% CI: 1.29–1.922), consistent with the primary estimate.

**Fig. 3:**
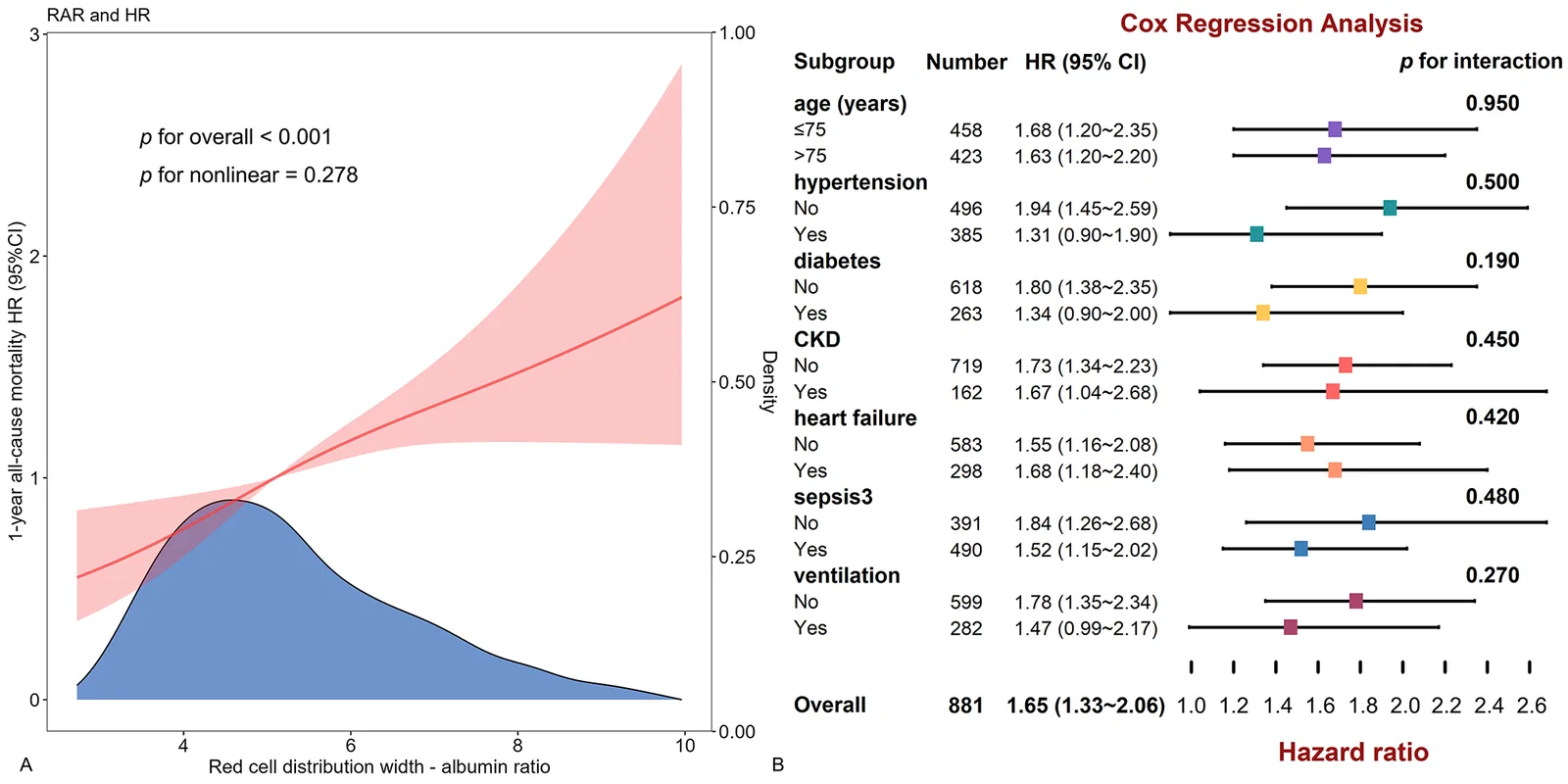
(**A**) Cubic spline model of the association between RAR and risk of 1-year all-cause mortality in model 3. (**B**) Subgroup analysis by age, hypertension, diabetes, CKD, heart failure, sepsis, and ventilation

Restricted cubic splines were fitted with three knots placed at the 5th, 50th, and 95th percentiles of the RAR distribution, following Harrell’s recommendation. The reference value was set at the median RAR. Thus, a positive and linear association was observed between RAR and HR for 1-year all-cause mortality (p for nonlinear > 0.05) (Fig. 3A). The solid line represents the estimated log hazard ratio and the shaded area denotes the 95% confidence interval.

**Table 2:** The association between red cell distribution width - albumin ratio and one-year all-cause mortality

RAR	*N*	model 0	E-value	model 1	E-value	model 2	E-value	model 3	E-value
cut-off value = 4.96		Hazard Ratio (Cox regression)							
Low (≤4.96)	409	reference	-	reference		reference		reference	
High (> 4.96)	472	2.19 (1.81, 2.65)	2.82(2.38)	1.95 (1.59, 2.38)	2.55(2.10)	1.83 (1.47, 2.27)	2.40(1.94)	1.65 (1.33, 2.06)	2.18(1.73)
Each unit increase	811	1.27 (1.20, 1.34)	-	1.25 (1.17, 1.32)	-	1.23 (1.15, 1.32)	-	1.17 (1.09, 1.25)	-

model 0: RAR was included; model 1: age, gender, diabetes, hypertension, AKI stage, CKD, heart failure, sepsis3, cirrhosis and COVID-19 were adjusted; model 2: WBC, hemoglobin, AST, total bilirubin, creatinine, BUN, anion gap, LDH, CRRT and ventilation were further adjusted; model 3: SOFA, APS Ⅲ, OASIS and CHARLSON were further adjusted. N: number of observation

### Subgroup analysis

Subgroup analysis with stratification was performed to investigate the association between RAR and 1-year all-cause mortality, along with an evaluation of potential interactions. The observed positive relationship remained consistent across subgroups, including age, hypertension, diabetes, CKD, heart failure, sepsis, and ventilation (all p for interaction > 0.05) (Fig. 3B). The aforementioned factors did not have a significant impact on the relationship between RAR and 1-year all-cause mortality.

### The value of predicting prognosis

**Fig. 4:**
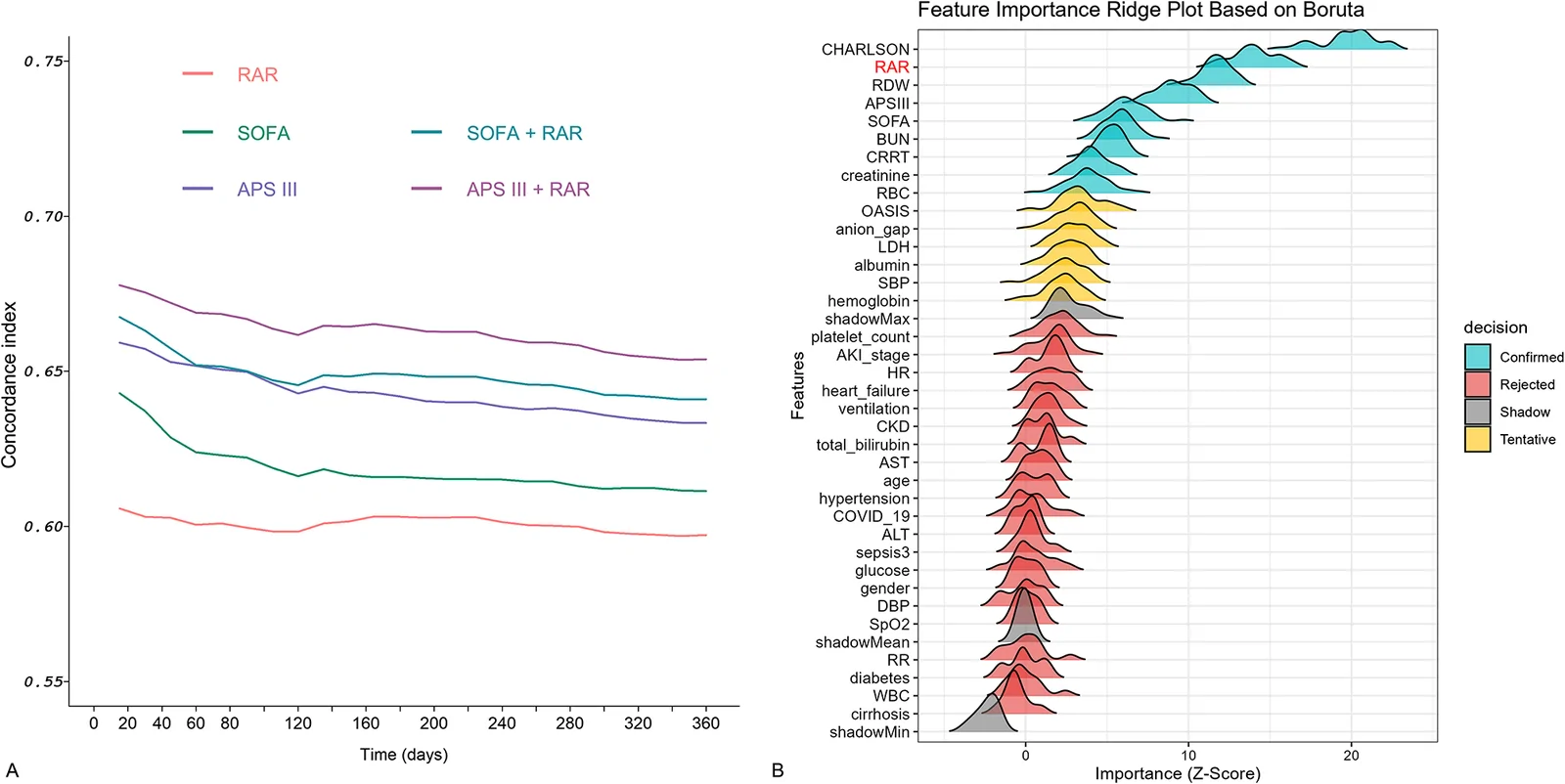
Concordance index predicted by RAR, SOFA, APS Ⅲ, SOFA plus RAR, APS Ⅲ plus RAR. The feature importance ridge plot based on the Boruta algorithm

Time-dependent analysis indicated that the inclusion of RAR significantly enhanced the prognostic value derived from SOFA and APS Ⅲ (Fig. 4A), and the concordance index increased from 0.610 to 0.640 for SOFA, from 0.632 to 0.653 for APS Ⅲ (*p* < 0.001 for both) (Supplementary Table 1). Bootstrap analysis (1000 resamples) confirmed that the C-index improvement was statistically significant: ΔC-index for SOFA + RAR vs. SOFA alone = 0.030 (95% CI 0.014–0.052, *P* < 0.001); ΔC-index for APS III + RAR vs. APS III alone = 0.021 (95% CI 0.008–0.041, *P* < 0.001).

Model performance was further evaluated by (i) the time-dependent area under the receiver operating characteristic curve (time-dependent AUC) for discrimination; (ii) calibration plots comparing predicted versus observed 1-year survival probabilities for calibration; and (iii) decision curve analysis (DCA) comparing net clinical benefit across threshold probabilities for models with and without RAR (Supplementary Figure. 3). We note that the Hosmer–Lemeshow test is designed for logistic regression and was therefore not applied to our Cox survival models; instead, calibration was assessed graphically using the standard calibration-curve approach for time-to-event data.

To assess the importance of predictors for 1-year all-cause mortality, the Boruta algorithm, based on random forest methodology, was utilized. For the Boruta analysis, the 1-year mortality event rate was 53.1%, and no additional class-balancing technique was applied. Random forests underlying the Boruta algorithm are relatively tolerant of moderate class imbalance, and feature importance was judged against shadow features rather than absolute classification accuracy, mitigating the impact of outcome imbalance on the ranking. The feature importance plot demonstrated that RAR emerged as a key contributor to outcome prediction, second only to the CCI (Fig. 4B).

To complement the Boruta importance ranking and clarify the direction and magnitude of each predictor’s contribution, SHAP (SHapley Additive exPlanations) values were computed for the fitted random-forest model. A SHAP summary (beeswarm) plot and a SHAP dependence plot illustrating all predictors were generated (Supplementary Figure. 2).

Exploratory mediation analysis: Because RAR is the ratio of RDW to albumin, decomposing its effect into pathways mediated by its own numerator and denominator does not fully satisfy standard mediation assumptions. The following analysis is therefore exploratory and hypothesis-generating only. Mediation analysis revealed that RDW exhibited a significant mediating effect (*P* = 0.010), while albumin demonstrated a masking effect (*P* = 0.044) between RAR and mortality risk. Nevertheless, RAR made a significant contribution to the total effect (*P* < 0.01 for both) (Supplementary Table 2, Supplementary Figure. 4).

### Sensitivity analysis

**Fig. 5:**
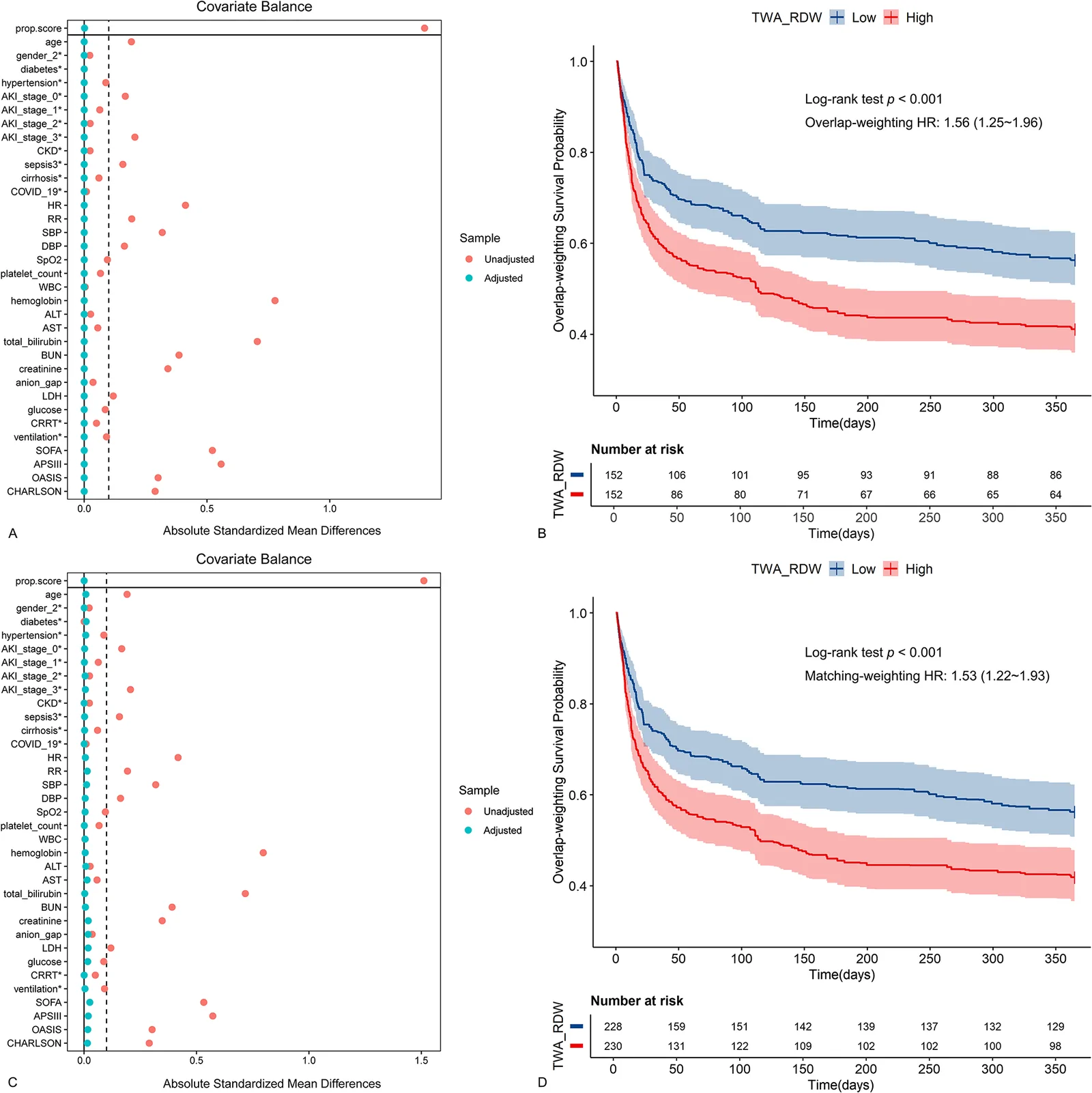
Absolute standardized mean differences before and after propensity score matching. (**A**: overlap-weighting, **C**: matching-weighting) Comparison of the cumulative survival probability between the high and low RAR groups after propensity score weighting. (**B**: overlap-weighting, **D**: matching-weighting)

To further assess the robustness of the findings, sensitivity analyses were conducted using two distinct propensity score weighting approaches. The use of overlap-weighting successfully balanced 34 baseline covariates between the two groups (absolute standardized mean difference < 0.1) (Fig. 5A), ensuring comparability for subsequent analysis. Survival analysis with overlap-weighting revealed that, compared to the low RAR group, the high RAR group had a 1-year all-cause mortality hazard ratio of 1.56 (95% CI:1.25–1.96, *p* < 0.001) (Fig. 5B). Similar conclusions were obtained when the match-weighting method was applied, with a hazard ratio (HR) of 1.53 (95% CI:1.22–1.93, *p* < 0.001) for high RAR compared to low RAR (Fig. 5C and D).

## Discussion

Our study suggests that an elevated RAR may serve as a potential adverse prognostic factor in breast cancer patients admitted to the ICU, particularly in resource-limited settings. The significant correlation between higher RAR levels and 1-year all-cause mortality persists even after controlling for confounding variables. Given its accessibility and low cost as part of routine hematological testing, RAR may represent a novel and reliable biomarker for identifying breast cancer patients in ICU at elevated risk of poor prognosis in clinical practice.

The RDW has recently been recognized as a potentially significant predictive and prognostic factor in breast cancer. An association was observed between increased RDW and a heightened risk of breast cancer, and RDW was proposed as a useful marker for differentiating malignant from benign breast lesions [17]. Elevated after-treatment RDW was associated with poor outcomes including overall survival (OS) and disease-free survival (DFS) in patients with breast cancer [18]. A study involving 1,783 patients demonstrated that relatively high preoperative RDW (> 13.5) and CA 15 − 3 levels (> 11.4) exhibited the strongest predictive value for mortality and recurrence, respectively, among hematological markers [19]. Yao et al. found that higher RDW levels were associated with larger tumor size and an increased incidence of lymph node metastasis, suggesting that RDW might serve as a surrogate indicator of tumor aggressiveness [20]. Moreover, elevated RDW was related not only with more advanced tumor stages but also with increased levels of inflammatory markers, highlighting the contribution of systemic inflammation to cancer prognosis [21]. As a simple and cost-effective prognostic marker for adverse outcomes in breast cancer patients, RDW facilitates early risk stratification and timely clinical intervention. In addition to RDW, serum albumin has been extensively investigated in relation to cancer prognosis. Hypoalbuminemia, defined as a reduced serum albumin concentration, often reflects poor nutritional status and systemic inflammation, both of which may contribute to increased morbidity, more frequent complications, and prolonged hospitalization [22]. Lower albumin levels have been correlated with more advanced disease stages and increased mortality in patients with colon and breast cancer [23–25]. These findings suggest that monitoring of albumin may offer valuable prognostic information, serving as a simple indicator of a patient’s overall health, nutritional condition, and risk of adverse clinical outcomes. Meanwhile, although we employed multivariable adjustment, propensity-score weighting, E-value quantification, and dose-response analysis–all of which support a robust association–the observational design precludes causal conclusions. Reverse causation (e.g., greater illness severity driving higher RAR rather than RAR causally increasing mortality) cannot be excluded. We therefore frame RAR as an independent prognostic marker rather than a proven causal driver of mortality. Future Mendelian randomization or prospective interventional studies would be required to establish causality.

The combination of RDW and albumin into a single metric, known as RAR, has attracted increasing interest as a potential prognostic indicator. Population-based studies demonstrated that a higher baseline RAR was associated with an increased risk of all-cause and cause-specific mortality [9, 10, 26], suggesting its universal role in inflammation-driven pathologies. However, studies specifically investigating the association between RAR and prognosis in breast cancer patients are currently lacking. Our study fills this gap in the literature. In this retrospective cohort study, breast cancer patients in ICU with a high RAR showed a significantly increased risk of 1-year all-cause mortality, with restricted cubic spline analysis confirming a dose-response relationship. The linear association between RAR and mortality may stem from its dual reflection of chronic inflammation (elevated RDW indicating oxidative stress [27] and immune dysfunction [28]) and malnutrition (hypoalbuminemia impairing immune response and tissue repair [29]). In breast cancer, systemic inflammation promotes tumor progression via NF-κB-mediated cytokine release [30], while hypoalbuminemia exacerbates chemotherapy toxicity and reduces treatment tolerance [31]. In critically ill breast cancer patients, several overlapping pathological processes may amplify the prognostic value of RAR. First, tumor-driven systemic inflammation elevates RDW through impaired erythropoiesis, increased oxidative stress, and chronic cytokine exposure. Second, cytotoxic chemotherapy and radiotherapy induce additional bone-marrow suppression and erythrocyte dysregulation, further increasing RDW beyond levels observed in cancer patients not receiving treatment. Third, the catabolic state of critical illness, compounded by frequent severe infections and evolving multi-organ dysfunction, accelerates hypoalbuminemia through increased capillary permeability, reduced hepatic synthesis, and heightened catabolism. By capturing these inflammatory, nutritional, and organ-dysfunction signals within a single ratio, RAR may reflect the cumulative physiological burden unique to this population–potentially explaining its strong prognostic performance relative to either component alone. In our study, we performed Cox regression analysis with adjustment for covariates, conducted subgroup analyses and calculated the E-value to minimize the potential impact of unmeasured confounding. We found that the adverse effect of RAR on prognosis was consistent. Moreover, our findings extend prior work in two directions. Population-based and ICU-based studies have linked elevated RAR to higher all-cause mortality across mixed malignancies and in general critically ill populations. A study reported that RAR was independently associated with mortality in a heterogeneous cancer ICU cohort; our study refines this observation by demonstrating a similar association specifically in breast cancer patients, with a fully adjusted HR of 1.65 that is broadly consistent with the magnitudes reported in other cancer types. Separately, studies in general (non-ICU) breast cancer cohorts have individually linked elevated RDW and hypoalbuminemia to poorer survival. Our work extends these observations by demonstrating the added value of combining these two markers into a single ratio and evaluating it within the specific clinical context of ICU admission, where acute physiological derangement may amplify the prognostic information carried by each component. Our results suggest that RAR may enhance the prognostic predictive value of the SOFA or APSⅢ score. To validate the robustness of the prognostic value of RAR, we further employed a ridge plot of feature importance and propensity score methods (overlap-weighting and matching-weighting) to confirm our findings. Based on these observations, we consider RAR to be a reliable prognostic factor in clinical practice.

This study has several notable strengths. First, to the best of our knowledge, no prior studies have specifically investigated the association between RAR and prognosis in breast cancer patients admitted to ICU, addressing an important and previously underexplored research gap. Second, we applied the feature importance ridge plot to quantify the prognostic contribution of RAR, a relatively underused yet informative approach in clinical prognostic modeling. However, several limitations should be acknowledged. A key limitation of this study is that the MIMIC-IV database does not record tumor-specific prognostic information, including TNM stage, molecular subtype (ER/PR/HER-2 status), and oncologic treatment regimens. These variables are likely associated with both RAR and mortality and may act as residual confounders. Although we mitigated this through multivariable adjustment, propensity-score weighting, and E-value quantification–demonstrating that an unmeasured confounder would need a risk ratio exceeding 2.18 to fully explain the observed association–bias from unmeasured oncologic factors cannot be excluded. Our findings should therefore be interpreted as associations rather than causal effects. Additionally, the patient population was derived from the MIMIC database and limited to breast cancer patients admitted to the ICU, which may limit the generalizability of our results to the broader breast cancer population. Furthermore, the absence of RDW and albumin means that only a portion of breast cancer patients are included in the analysis, which introduces potential selection bias. Although we attempted external validation using the eICU Collaborative Research Database (eICU-CRD), the limited number of eligible patients reduced the statistical power of the analysis. In that analysis in the eICU-CRD (*n* = 242), the hazard ratio for high versus low RAR was 1.11 (95% CI: 0.89 to 1.37); this analysis was underpowered and should be regarded as hypothesis-generating. Validation in larger, prospective, multicenter cohorts–ideally including offline real-world data–remains essential to establish broader applicability. Future studies could further investigate longitudinal trajectories of biomarkers [32].

## Conclusion

In ICU-admitted breast cancer patients, a higher RAR was independently associated with increased 1-year all-cause mortality. As a routine laboratory parameter, RAR may aid risk stratification; however, prospective studies incorporating tumor stage, molecular subtype, and treatment data are needed to confirm and extend these associations.

## Supplementary Information

Below is the link to the electronic supplementary material.

Supplementary Material 1

Supplementary Material 2

Supplementary Material 3

Supplementary Material 4

Supplementary Material 5

## Data Availability

The datasets presented in this study can be obtained from MIMIC database on the premise of completing its training ( https://physionet.org/content/mimiciv/3.1/)

## References

[1] Sung H, et al. Global Cancer Statistics 2020: GLOBOCAN Estimates of Incidence and Mortality Worldwide for 36 Cancers in 185 Countries. CA Cancer J Clin. 2021;71:209–49. 10.3322/caac.21660 33538338

[2] Schubbe D, Yen RW, Durand MA. How Does Patient Socioeconomic Position Affect Breast Cancer Surgical Treatment and Mortality? A Rapid Review. Breast Cancer (Dove Med Press. 2021;13:595–601. 10.2147/BCTT.S293635 PMC8558100 34737634

[3] Kim KM, Nerlekar R, Tranah GJ, Browner WS, Cummings SR. Higher red cell distribution width and poorer hospitalization-related outcomes in elderly patients. J Am Geriatr Soc. 2022;70:2354–62. 10.1111/jgs.17819 35506925

[4] Conway R, Byrne D, O’Riordan D, Silke B. Red cell distribution width as a prognostic indicator in acute medical admissions. 10.3390/jcm12165424 PMC10455471 37629466

[5] Peng S, Li W, Ke W. Association between red blood cell distribution width and all-cause mortality in unselected critically ill patients: Analysis of the MIMIC-III database. Front Med (Lausanne). 2023;10:1152058. 10.3389/fmed.2023.1152058 PMC10098131 37064043

[6] Owusuaa C, Dijkland SA, Nieboer D, van der Heide A. C. C. D. van der Rijt, Predictors of mortality in patients with advanced cancer-: a systematic review and meta-analysis. Cancers (Basel). 2022;14. 10.3390/cancers14020328 PMC8774229 35053493

[7] Hanson LC, et al. Mortality Risk for Patients With Stage IV Cancer and Acute Illness Hospitalization. J Pain Symptom Manage. 2021;61:797–804. 10.1016/j.jpainsymman.2020.10.015 PMC8075274 33096216

[8] Kühn T, et al. Albumin, bilirubin, uric acid and cancer risk: results from a prospective population-based study. Br J Cancer. 2017;117:1572–9. 10.1038/bjc.2017.313 PMC5680462 28898231

[9] Hao M, et al. Ratio of Red Blood Cell Distribution Width to Albumin Level and Risk of Mortality. JAMA Netw Open. 2024;7:e2413213. 10.1001/jamanetworkopen.2024.13213 PMC11134218 38805227

[10] Lu C, et al. Red blood cell distribution width-to-albumin ratio is associated with all-cause mortality in cancer patients. J Clin Lab Anal. 2022;36:e24423. 10.1002/jcla.24423 PMC9102686 35396747

[11] Yuan Q, et al. Machine learning and SHAP value interpretation for predicting the response to neoadjuvant chemotherapy and long-term clinical outcomes in Chinese female breast cancer. Ann Med. 2025;57:2541316. 10.1080/07853890.2025.2541316 PMC12322993 40754951

[12] Ye R, et al. Identification of the shared gene signatures in retinoblastoma and osteosarcoma by machine learning. Sci Rep. 2024;14:31355. 10.1038/s41598-024-82789-7 PMC11682156 39733097

[13] Yuan Q, et al. Machine learning-based identification of kbhb-affected tumor cell subsets as prognostic and therapeutic targets in breast cancer. J Transl Med. 2025;24:71. 10.1186/s12967-025-07555-3 PMC12802299 41382084

[14] Lou J, et al. The non-linear association between creatinine-to-albumin ratio and medium-term mortality in patients with sepsis accompanied by acute kidney injury in the intensive care unit: a retrospective study based on the MIMIC database and external validation. Front Cell Infect Microbiol. 2025;15:1602921. 10.3389/fcimb.2025.1602921 PMC12715007 41425961

[15] Lou J, et al. Association between serum glucose potassium ratio and short- and long-term all-cause mortality in patients with sepsis admitted to the intensive care unit: a retrospective analysis based on the MIMIC-IV database. Front Endocrinol (Lausanne). 2025;16:1555082. 10.3389/fendo.2025.1555082 PMC12343221 40810063

[16] Mansournia MA, Nazemipour M. Recommendations for accurate reporting in medical research statistics. Lancet. 2024;403:611–2. 10.1016/S0140-6736(24)00139-9 38368003

[17] Lu F, Pan S, Qi Y, Li X, Wang J. The clinical application value of RDW, CA153, and MPV in breast cancer. Clin Lab. 2021;67(6). 10.7754/Clin.Lab.2020.200507 33616344

[18] Lee HS, et al. The usefulness of red blood cell distribution width and its ratio with platelet count in breast cancer after surgery and adjuvant treatment: a retrospective study. Gland Surg. 2022;11:1864–73. 10.21037/gs-22-410 PMC9840999 36654946

[19] Yoo YC, Kim JM, Park JY, et al. Preoperative routine laboratory markers for predicting postoperative recurrence and death in patients with breast cancer. J Clin Med. 2021;10(19):4499. 10.3390/jcm10122610 PMC8231951 34199276

[20] Yao D, Wang Z, Cai H, Li Y, Li B. Relationship between red cell distribution width and prognosis in patients with breast cancer after operation: a retrospective cohort study. Biosci Rep. 2019;39(7). 10.1042/BSR20190740 PMC6629944 31262969

[21] Xiao J, et al. Association between red cell distribution width and all-cause mortality in patients with breast cancer: A retrospective analysis using MIMIC-IV 2.0. PLoS ONE. 2024;19:e0302414. 10.1371/journal.pone.0302414 PMC11095716 38748743

[22] Christina NM, et al. Hypoalbuminemia and colorectal cancer patients: Any correlation? A systematic review and meta-analysis. Med (Baltim). 2023;102:e32938. 10.1097/MD.0000000000032938 PMC10162791 36827017

[23] Haskins IN, Baginsky M, Amdur RL, Agarwal S. Preoperative hypoalbuminemia is associated with worse outcomes in colon cancer patients. Clin Nutr. 2017;36:1333–8. 10.1016/j.clnu.2016.08.023 27612919

[24] Di Castelnuovo A, et al, et al. The association between hypoalbuminemia and risk of death due to cancer and vascular disease in individuals aged 65 years and older: findings from the prospective Moli-sani cohort study. EClinicalMedicine. 2024;72:102627. 10.1016/j.eclinm.2024.102627 PMC11247150 39010980

[25] Xiang M, et al. Low serum albumin levels and high neutrophil counts are predictive of a poorer prognosis in patients with metastatic breast cancer. Oncol Lett. 2022;24:432. 10.3892/ol.2022.13552 PMC9608081 36311691

[26] Xu W, et al. Association between red blood cell distribution width to albumin ratio and prognosis of patients with sepsis: A retrospective cohort study. Front Nutr. 2022;9:1019502. 10.3389/fnut.2022.1019502 PMC9539557 36211519

[27] Joosse HJ, et al. In-vitro and in-silico evidence for oxidative stress as drivers for RDW. Sci Rep. 2023;13:9223. 10.1038/s41598-023-36514-5 PMC10247684 37286717

[28] Wang J, et al. APACHE-II, and SOFA Scores Were a Possible Cause of 28-Day Mortality in Sepsis Patients. Infect Drug Resist. 2024;17:1199–213. Immune Dysfunction-Associated Elevated. 10.2147/IDR.S442169 PMC10981425 38560707

[29] Soeters PB, Wolfe RR, Shenkin A. Hypoalbuminemia: Pathogenesis and Clinical Significance. JPEN J Parenter Enter Nutr. 2019;43:181–93. 10.1002/jpen.1451 PMC7379941 30288759

[30] Pavitra E, et al. The role of NF-κB in breast cancer initiation, growth, metastasis, and resistance to chemotherapy. Biomed Pharmacother. 2023;163:114822. 10.1016/j.biopha.2023.114822 37146418

[31] Hua Y, et al. Predictive model of chemotherapy-related toxicity in elderly Chinese cancer patients. Front Pharmacol. 2023;14:1158421. 10.3389/fphar.2023.1158421 PMC10169599 37180715

[32] Lou J, et al. Longitudinal biomarker trajectories and their prognostic utility for 21-day mortality in burn patients with sepsis: a retrospective cohort study. Inflamm Res. 2026;75:20. 10.1007/s00011-025-02178-2 PMC12823687 41563442

