# Supplementary material for: Red blood cell distribution width-to-albumin ratio as a novel predictor for mortality in breast cancer patients admitted to ICU: a retrospective analysis using MIMIC-IV 3.1
Source: BMC Med Inform Decis Mak. 2026 Jul 13;26:394. doi: 10.1186/s12911-026-03711-x (PMC13644060; doi:10.1186/s12911-026-03711-x)
Supplement: Supplementary file 1 [file 12911_2026_3711_MOESM4_ESM.doc]

**Supplementary Table 1**. Concordance index for models in predicting the all-cause mortality.

| Models | Concordance index | *p* value |
| --- | --- | --- |
| RAR | 0.597 (0.573, 0.620) | - |
| SOFA | 0.610 (0.584, 0.637) | < 0.001 |
| SOFA + RAR | 0.640 (0.613, 0.666) |  |
| APS Ⅲ | 0.632 (0.605, 0.659) | < 0.001 |
| APS Ⅲ + RAR | 0.653 (0.626, 0.679) |  |

**Supplementary Table 2**. Mediation analysis with confidence intervals from bootstrap (1000 resamples).

| Mediators | Effect | Estimate | 95% CI | *p* value |
| --- | --- | --- | --- | --- |
| RDW | ACME (average) | -12111 | −74222 ~ −228 | 0.010 |
|  | ADE (average) | -16232 | −93237 ~ 8.76 | 0.007 |
|  | Prop. Mediated (average) | 42.0% | 9.2% ~ 100% | 0.010 |
| Albumin | ACME (average) | 12785 | 59 ~ 72895 | 0.044 |
|  | ADE (average) | -34858 | -221170 ~ -1372 | < 0.001 |
|  | Prop. Mediated (average, masking) | 36.7% | 7.6% ~ 61.4% | < 0.001 |
